# Physicochemical Analysis of Mixed Venous and Arterial Blood Acid-Base State in Horses at Core Temperature during and after Moderate-Intensity Exercise

**DOI:** 10.3390/ani12151875

**Published:** 2022-07-22

**Authors:** Michael I. Lindinger, Amanda P. Waller

**Affiliations:** 1 Research and Development, The Nutraceutical Alliance Inc., Guelph, ON N1E 2G7, Canada; 2 Center for Clinical & Translational Research, Nationwide Children’s Hospital, Columbus, OH 43205, USA; amanda.waller@nationwidechildrens.org

**Keywords:** stewart acid-base, strong ion difference, SID, total weak acid concentration, A_tot_, acid-base partitioning, physicochemical approach, pulmonary gas exchange, CO_2_ production, ventilation

## Abstract

**Simple Summary:**

The purposes of the present study were to determine the effect of core body temperature on acid-base variables and determine the origins of acid-base changes in the arterial and mixed venous blood of horses during exercise and recovery. Moderate intensity exercise resulted in an increase in body temperature that, in addition to exercise, affected acid-base status and gas partial pressures. Moderate intensity exercise resulted in a mild alkalosis that had markedly different origins in arterial blood than in mixed venous blood, and this was affected by the increase in core temperature during exercise and its resolution during recovery. In order to fully understand how acid-base status changes during exercise and recovery, it is importance to quantify the changes in both arterial and mixed venous blood, with adjustment to core temperature. Acid-base assessments using jugular vein blood samples are limited in comparison.

**Abstract:**

The present study determined the independent contributions of temperature, strong ion difference ([SID]), total weak acid concentration ([A_tot_]) and PCO_2_ to changes in arterial and mixed venous [H^+^] and total carbon dioxide concentration ([TCO_2_]) during 37 min of moderate intensity exercise (~50% of heart rate max) and the first 60 min of recovery. Six horses were fitted with indwelling carotid and pulmonary artery (PA) catheters, had PA temperature measured, and had blood samples withdrawn for immediate analysis of plasma ion and gas concentrations. The increase in core temperature during exercise (+4.5 °C; *p* < 0.001) significantly (*p* < 0.05) increased PO_2_, PCO_2_, and [H^+^], but without a significant effect on [TCO_2_] (*p* > 0.01). The physicochemical acid-base approach was used to determine contributions of independent variables (except temperature) to the changes in [H^+^] and [TCO_2_]. In both arterial and venous blood, there was no acidosis during exercise and recovery despite significant (*p* < 0.05) increases in [lactate] and in venous PCO_2_. In arterial blood plasma, a mild alkalosis with exercise was due to primarily to a decrease in PCO_2_ (*p* < 0.05) and an increase in [SID] (*p* < 0.1). In venous blood plasma, a near absence of change in [H^+^] was due to the acidifying effects of increased PCO_2_ (*p* < 0.01) being offset by the alkalizing effects of increased [SID] (*p* < 0.05). The effect of temperature on PO_2_ (*p* < 0.001) resulted in an increased arterio-venous PO_2_ difference (*p* < 0.001) that would facilitate O_2_ transfer to contracting muscle. The simultaneous changes in the PCO_2_ and the concentrations of the other independent acid-base variables (contributions from individual strong and weak ions as manifest in [SID] and [A_tot_]) show complex, multilevel control of acid-base states in horses performing even moderate intensity exercise. Correction of acid-base variables to core body temperature presents a markedly different physiological response to exercise than that provided by variables measured and presented at an instrument temperature of 37 °C.

## 1. Introduction

Exercise in the horse, as in other animals, results in a number of integrated physiological effects and responses that involve muscular, circulatory, respiratory, endocrine, immune and neural systems. While different breeds engage in different types of activities, and these may be associated with some differences in responses [1], there are similarities in the adaptive responses to exercise [2]. For example, whole-body acid-base balance is dependent on the integrated responses of the muscular, respiratory, vascular, hepatic, cutaneous, renal and gastrointestinal systems. Significant acid-base disturbances are often associated with moderate- to high-intensity exercise and prolonged-duration exercise, as well as with many pathologies. Within the field of exercise physiology, acid-base balance is particularly important because of the association between acidification and muscle fatigue [3].

There are numerous descriptive studies of acid-base balance in horses in response to different types of exercise [4,5,6,7], and as reviewed previously [8]. However, only a handful of studies have attempted to take a mechanistic approach to determine the physiological and physicochemical mechanisms that underly changes in plasma H^+^ and TCO_2_ concentrations [9,10,11,12,13]. Most studies are also hindered by the fact that jugular venous blood was sampled as the only blood source for analysis, and thus does not represent mixed venous blood nor arterial blood. While the brain and head of the horse are metabolically active during exercise, cranial metabolism is not representative of the metabolism of contracting muscles [14]. Given that: (1) nearly all muscles in the exercising horse are undergoing contractile activity, and that muscle mass comprises ~40% of lean body mass [15]; (2) blood flow to muscles is increased several fold; and (3) blood flow to most non-contracting tissues is reduced during exercise [16,17], a sample of mixed venous blood provides for a more accurate analysis of whole body acid-base state. This is especially true when coupled with analysis of arterial blood obtained after perfusion of the lungs but prior to perfusion of tissues. Differences between arterial and mixed venous blood represent the contributions of the metabolic activities of all of the tissues, and additionally the contribution of the lungs can also be assessed [9,10]. Unfortunately there exists minimal data on acid-base or blood gas alterations that may occur in arterial and/or mixed venous blood during exercise, owing to the difficulty of repeatedly obtaining such samples. Finally, the majority of studies examining equine acid-base responses during exercise either did not use temperature adjustments or adjusted to rectal temperatures (T_re_), which is an inaccurate surrogate for core body temperature in exercising equids [11,18].

The descriptive studies of acid-base balance in horses are helpful to those people that only sample jugular venous blood, which includes most veterinarians and researchers. Even so, many researchers and veterinarians have not been satisfied with the simplified metabolic/respiratory acid-base interpretation arising from the work of the traditional acid-base approach [9,19,20]. Thus, there has been an interest in using the physicochemical approaches outlined by Henderson [21], Singer and Hastings [22], and most notably Stewart [23,24] to provide a more mechanistic approach to understanding the origins of acid-base disturbances and the physiological mechanisms that underlie their correction [19,25,26,27,28]. In addition, the acid-base state in animals is complicated by the effects of temperature on the blood/tissue pH, PCO_2_ and the PO_2_ [29,30,31,32]. While instrument-measured (at 37 °C) values for these variables are routinely employed, they are not the values in vivo. It is the in vivo values of the variables that are being sensed with respect to ventilatory control, tissue gas exchange and acid-base regulation. Notably, core body temperature may increase by 4 °C or more with moderate to high intensity exercise, and this, independent of changes in the “independent” acid-base variables [SID], PCO_2_ and [A_tot_], results in a PCO_2_ that is increased by ~6 mm Hg and a pH that is decreased by 0.02 units ([H^+^] increased by ~1.4 nmoles/L). These temperature effects may manifest in increased [HCO_3_^−^] and [TCO_2_] [30,33].

There is a dearth of comprehensive, mechanistic studies of acid-base balance in exercising horses where blood has been sampled from an artery and from the mixed venous circulation. The most complete analysis of acid-base responses during exercise in equids is limited to: (1) Forster et al. [34] who measured, using ponies, responses in arterial and mixed venous blood during short-duration, submaximal exercise resulting in steady-state within 7 min; and (2) Vengust et al. [10], who used Standardbred horses during a high intensity incremental exercise test to fatigue (occurred within 5 min) and into 15 min of recovery. Importantly, both studies temperature-adjusted the plasma gas and pH values to that of the horse during the exercise and they used a physicochemical approach to determine the contributions of changes in acid-base state, which they characterized in both arterial and mixed venous plasma.

Thus, the purpose of this paper is to present a detailed time-course of acid-base changes with partitioning of the contributions of independent acid-base variables to the changes in [H^+^] and [TCO_2_] in both arterial and mixed venous blood for 30^+^ min of steady-state intensity exercise and for 60 min of post-exercise recovery. Importantly, this study used temperature adjustments for arterial and mixed venous blood and extended the physicochemical analyses performed in previous studies. In addition, the sampling of both mixed venous and arterial blood from horses at rest, during moderate intensity exercise and during recovery, provided the benefits of determining contributions of contracting/recovering tissues as well as the lungs to acid-base balance during exercise and recovery. It was hypothesized that during exercise: (1) decreases in [SID] and [A_tot_] would be the greatest contributors to the decreases in plasma pH and [HCO_3_^−^] in both mixed venous and arterial blood; (2) increased PCO_2_ contributes significantly to the acidosis in mixed venous blood; (3) whereas decreased PCO_2_ contributes significantly to an alkalizing effect in arterial blood; and (4) adjustment of acid-base parameters to real time data of core body temperature will provide crucial distinctions in the interpretation of blood gas measurements, which greatly improve the accuracy of the reported results.

## 2. Methods

### 2.1. Ethical Approval

The care and use of animals for this research were approved by the University of Guelph Animal Care Committee and the experiments conducted in compliance with the university’s animal care policy and in accordance with the guidelines of the Canadian Council on Animal Care.

### 2.2. Animals

This is the same study and horses as represented by these previous publications, where exercise duration was limited by pulmonary artery blood temperature reaching 41.5 °C [35,36,37].

Six thoroughbred horses with age 3–6 years and body weight 435–470 kg were studied. The horses were housed indoors and fed a diet of grass hay and mixed grain, with water and a trace mineral/salt block available ad libitum. All horses had the right carotid artery surgically relocated to the subcutaneous position at least 2 months prior to the start of the study.

Leading up to the experiments, the horses were conditioned 5 days per week for 12 weeks with a progressive program of walking, trotting, cantering and galloping on an indoor high-speed treadmill (Säto, Sweden) to achieve a steady-state level of fitness. The maximal O_2_ uptake of each horse was subsequently determined on two separate occasions [37], and the mean VO_2max_ of all horses was 139 ± 5.8 mL·kg^−1^·min^−1^ prior to the start of experiments. At the end of the study all horses were returned to the university research herd.

Catheterization of the raised carotid artery and a jugular vein was performed after aseptic preparation and local analgesia of the skin. Blood temperature was measured by inserting a thermocouple into the pulmonary artery within an 8-Fr polyethylene catheter. The catheter was introduced via a jugular vein, and its position within the pulmonary artery was verified by pressure wave recordings.

### 2.3. Exercise Protocol

Horses completed the exercise protocol on a treadmill in a room in which temperature (22 ± 1 °C) and relative humidity (50 ± 3%) could be controlled. Food and water were withheld for 3 h prior to, and for the duration of, each experiment. Body mass was measured on a large animal scale (±0.5 kg, Marsh Instruments, Mississauga, ON, Canada) immediately prior to the exercise protocol and at 30 min of recovery following exercise.

Exercise was conducted on a treadmill set on a 10% slope with a high-speed fan providing air movement with a velocity of 2 m/s. The fan was situated in front of the horse and directed air over the anterior and dorsal aspects of the horse. Resting measurements were obtained during a 30 min period prior to exercise during which the horses remained stationary on the treadmill. The exercise test consisted of a 5 min warm-up (1.5 m/s) followed by exercise at a speed calculated by regression analysis to elicit 50% of individual peak VO_2_ (range 3.8–4.3 m/s). Exercise was continued until attainment of PA blood temperature of 41.5 °C. After the first 5 min of recovery the horses walked at a slow pace for the next 25 min, then stood quietly for a further 30 min.

Blood samples (approximately 8 mL) were obtained in 10 mL syringes containing 100 IU of lithium heparin (Sarstedt, Nűmbrecht, Germany) at rest, 2, 5, 15, and 30 min and at end of exercise and at 2, 5, 15, 30 and 60 min of recovery.

### 2.4. Analysis

Blood gases and plasma ion concentrations were measured using a Statprofile 5 ion analyzer (Nova Biomedical Canada Ltd., Mississauga, ON, Canada). Plasma was analyzed for protein with a clinical refractometer (model SPR-T2, Atago, Japan).

### 2.5. Calculations

The detailed acid-base analysis that permits the determination of the contributions of independent variables (partitioning) to the changes in the concentrations of dependent acid-base variables is that employed previously [38] and adapted for use in horses [11,39].

Plasma pH was adjusted for temperature, because the dissociation of water is increased when temperature is increased, resulting in increased [H^+^]. The relationship between pH and temperature in plasma and whole blood is complex and influenced by the hemoglobin content, plasma protein concentration and plasma CO_2_ content [30]. However, temperature adjusted pH can be estimated within ±5% [32,40] using the equation provided by Ashwood et al. [31] and used by Taylor et al. [33], but with the square brackets in the correct locations as shown here:

pH_adjusted_ = pH_m_ + [−0.0147 + 0.0065 × (7.400 − pH_m_)] (T_core_ − 37) (1)

where pH_m_ is the instrument measured pH at 37 °C and T_core_ is the temperature measured in the pulmonary artery at that time point [29,30].

Plasma PCO_2_ was also adjusted for temperature, because the solubility coefficient is also dependent on temperature [41], using the equation from Taylor et al. [33]:

PCO_2(adjusted)_ = PCO_2_ × e^(0.04375(T_core_^−37))^^ (2)

where T is temperature in °C. In order to verify the accuracy of the estimates, the temperature adjusted values for pH and PCO_2_ were directly compared with the plots provided by Reeves ([30]; see his Figures 4 and 5), with which they were in perfect agreement.

In order to calculate the [HCO_3_^−^] adjusted for temperature using the Henderson–Hasselbalch equation, one must first calculate the temperature-adjusted first apparent dissociation constant for carbonic acid pK′_1(adjusted)_ as described by Rispens et al. [42]:

(3) $${pK\prime }_{1\left(\mathrm{adjusted}\right)}=-4.7416+\frac{1840.141}{T}+0.015906T-\log(1+\frac{0.020682}{10^{\left(-\mathrm{pH}+7\right)}})$$

where T is temperature in ^o^*K*. [HCO_3_^−^]_adjusted_ was calculated as:

(4) $$\left[{\mathrm{HCO}}_{3}{}^{-}]{}_{\mathrm{adjusted}}=\left({K\prime }_{1\left(\mathrm{adjusted}\right)}\times S\times {\;\mathrm{PCO}}_{2}\right)/[H^{+}\right]$$

where [H^+^] is calculated from pH_adjusted_, PCO_2_ is PCO_2(adjusted)_ calculated as described in equation 2, K′_1_ is 10^−pK’1(adjusted)^ where pK′_1_ was calculated as described in Equation (3), and S (the CO_2_ solubility coefficient) is calculated as described in Equation (5) using the data of Austin et al. [41]:

S = 0.0797−(0.00206 × T) + (0.0000200 × T^2^) (5)

Temperature for adjustment for PO_2_ was performed using the equation provided by Taylor [33]:

PCO_2(adjusted)_ = PCO_2(37)_ × 10^[0.021 × (T−37)]^

Temperature adjustment for PO_2_ was performed using the equation provided by Taylor [33] using equations published previously [43,44].

PO_2(adjusted)_ = PO_2_ × e [2.303 (T − 37) × {5.49 × 10^−11^)y + 0.071}/{9.72 × 10^−9^)y + 2.30}] (6)

where y = e^3.88 × ln(PO_2_^)^^.

Measured and temperature adjusted data were analyzed using Acid-Basics II software (P.D. Watson, U. South Carolina) using the ‘Stewart model’ for ‘buffers’. The software uses an iterative process to compute the best-fit values of dependent acid-base variables using the equation from Stewart [23]:

[H^+^] + (K_A_ + [SID]) [H^+^]^3^ + {K_A_ ([SID] − [A_tot_]) − (K_C_ × pCO_2_ + K_W_)} [H^+^]^2^ − {K_A_ (K_C_ × pCO_2_ + K_W_) + (K_3_ × K_C_ × pCO_2_)} [H^+^] − K_A_ × K_3_ × K_C_ × pCO_2_) = 0 (7)

where K_W_, K_A_, K_3_ and K_C_ are the equilibrium constants for dissociations of water, total weak acids, carbonic acid and bicarbonate, respectively. The default values for some of these “constants” did not yield best fits. Therefore, the K_A_ for total weak acids, and the anion equivalency of plasma proteins, both of which are somewhat variable [25,38,45,46], were manipulated iteratively [45] so as to yield a best fit of the calculated pH, [H^+^] and [HCO_3_^−^] relative to their measured values.

Manipulation of the anion equivalency of plasma weak acids (includes contributions from both protein and inorganic phosphate) showed that 0.22 mEq/g protein (similar to Staempfli—0.21; Constable—0.224) provided the best fit [45]. A K_A_ of 1.1 × 10^−7^ (Eq/L) was used because this resulted in the best fit when using all of the data, with no difference between environmental conditions. The K_A_ of 2.21 × 10^−7^ Eq/L [46] or 2.22 × 10^−7^ Eq/L determined by Constable [45] did not provide a best fit of the data. A K_3_ of 6 × 10^−11^ Eq/L was used in order to keep this constant between published papers, despite the software calling for a more accurate K_3_ of 5.76 × 10^−11^ Eq/L (Acid-Basics II software). The default value for the CO_2_ solubility coefficient was changed from 0.0309 to 0.0300, the experimentally determined mean of the temperature adjusted data.

### 2.6. Statistics

The data were analyzed using parametric tests following statistical verification of normal distribution of data. Due to missing data arising from the occasional inability to obtain a blood sample or with measuring electrode malfunction, the final design was not balanced, requiring comparisons to be made using two-way ANOVA (condition and time) without repeated measures. When a significant F-ratio was obtained, then the Bonferroni post hoc test was performed, which handles missing data well. Within condition, and within time, series of one-way repeated measures ANOVA were performed to compare means, again using the Bonferroni post hoc test. Statistical significance was accepted at *p* < 0.05 with power > 0.8. Values are presented as mean ± SD and with minimum and maximum values.

## 3. Results

The changes in some of the blood [35] as well as thermal and cardiorespiratory [37] parameters for this study have been reported previously.

### 3.1. Temperature-Dependent Acid-Base Variables

**Figure 1:**
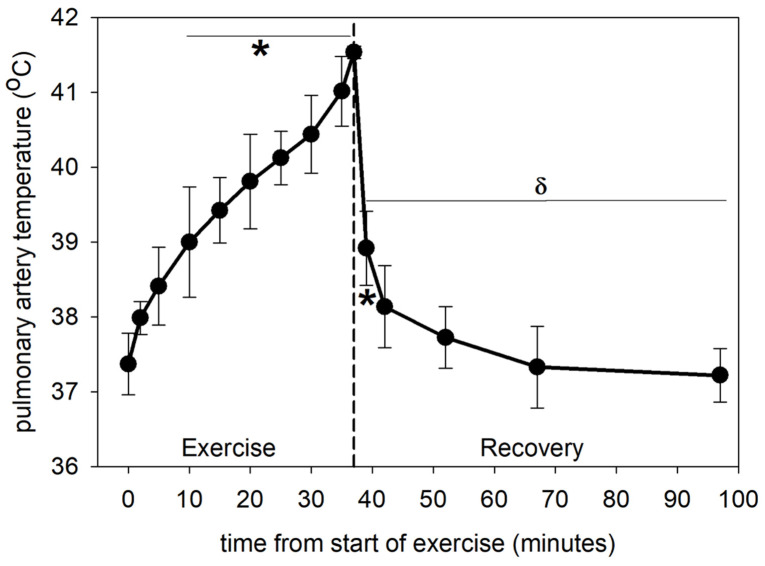
Core (pulmonary artery) temperature at rest before exercise (time = 0), during exercise and recovery from exercise. * significantly different (*p* < 0.05) from time 0. δ significantly different (*p* < 0.001) from end of exercise (37 min). Data from 6 horses, mean ± SD.

Horses completed 37.0 ± 2.0 min of exercise prior to reaching a PA blood temperature of 41.5 °C. Recovery of PA blood temperature was initially very rapid and reached values not different from pre-exercise rest at 5 min of recovery (Figure 1). These data have been reported previously [39] and are presented here for convenience.

**Figure 2:**
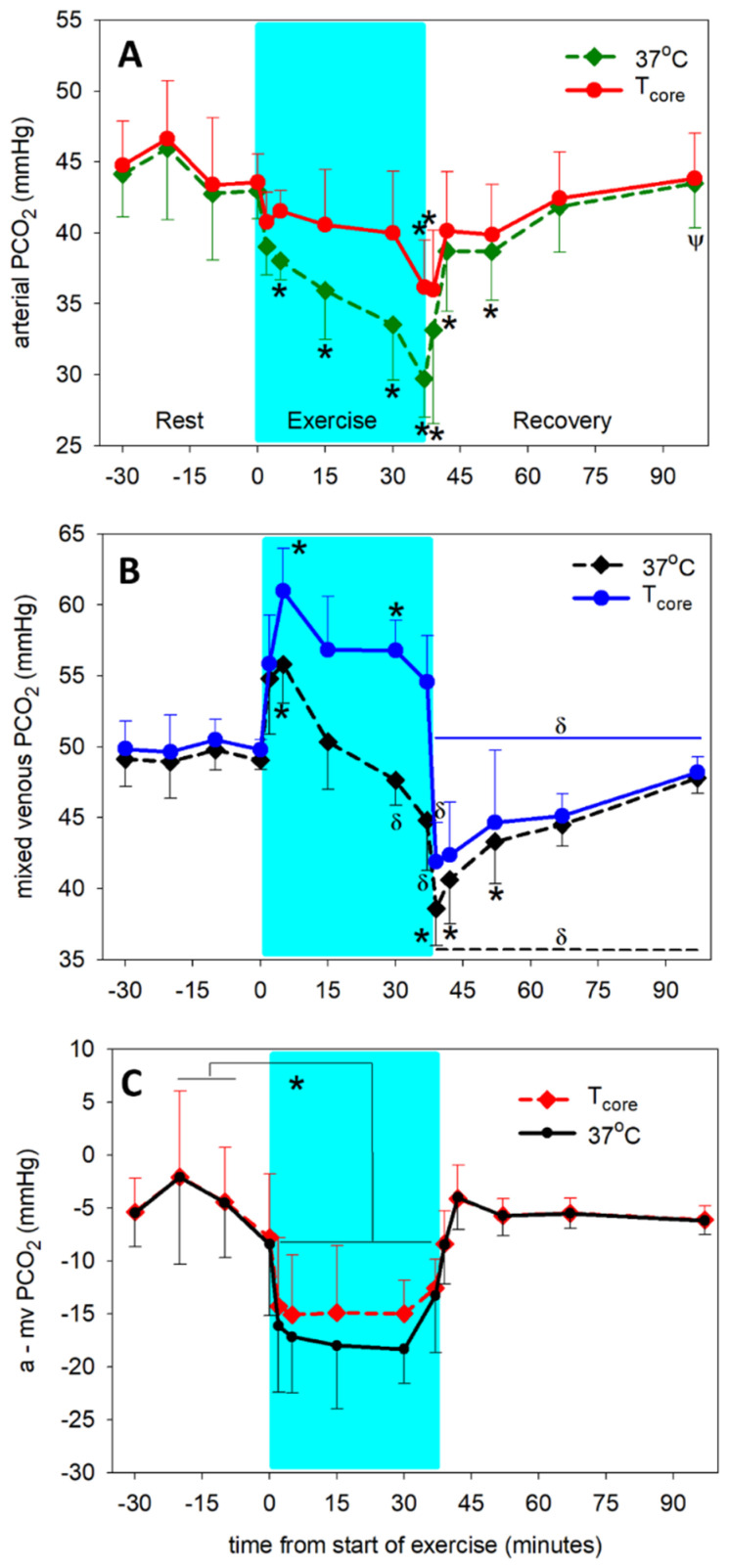
Time course of arterial (**A**) and mixed venous (mv, (**B**)) PCO_2_ and a-mv PCO_2_ difference (**C**) at rest prior to exercise, during exercise (shaded) and recovery from exercise. Dashed lines: measured at 37 °C. Solid lines: adjusted for core (pulmonary artery) temperature. * significantly different (*p* < 0.001) from time 0. ψ significantly different (*p* < 0.001) from end of exercise (37 min). δ significantly different (*p* < 0.001) from 5 min of exercise). Data from 6 horses, mean ± SD.

When measured at 37 °C, arterial plasma PCO_2_ decreased (*p* < 0.001) by 5 min of exercise and continued to decrease by ~14 mmHg by the end of exercise, returning to values not different from pre-exercise by 5 min of recovery (Figure 2A). Venous plasma PCO_2_ increased (*p* < 0.001) rapidly with the onset of exercise, peaking at 5 min and then progressively decreased (*p* < 0.001) to below resting values at the end of exercise (Figure 2B). Venous PCO_2_ remained below pre-exercise values (*p* < 0.001) until after 15 min of recovery.

Temperature adjustment of PCO_2_ significantly increased arterial and mv PCO_2_ values during the entire exercise period. During the pre-exercise and recovery periods, there was no difference between plasma PCO_2_ measured at 37 °C versus adjusted to PA blood temperature (T_core_). In arterial plasma, the PCO_2_ at T_core_ was decreased (*p* < 0.001) only at the end of exercise and 2 min of recovery compared to pre-exercise (Figure 2A). In mv plasma, PCO_2_ at T_core_ was elevated (*p* < 0.001) at 5 and 30 min of exercise, then decreased (*p* < 0.001) to below pre-exercise values during the recovery period (Figure 2B). Temperature adjustment of the PCO_2_ did not significantly affect the a-mv PCO_2_ difference (Figure 2C).

**Figure 3:**
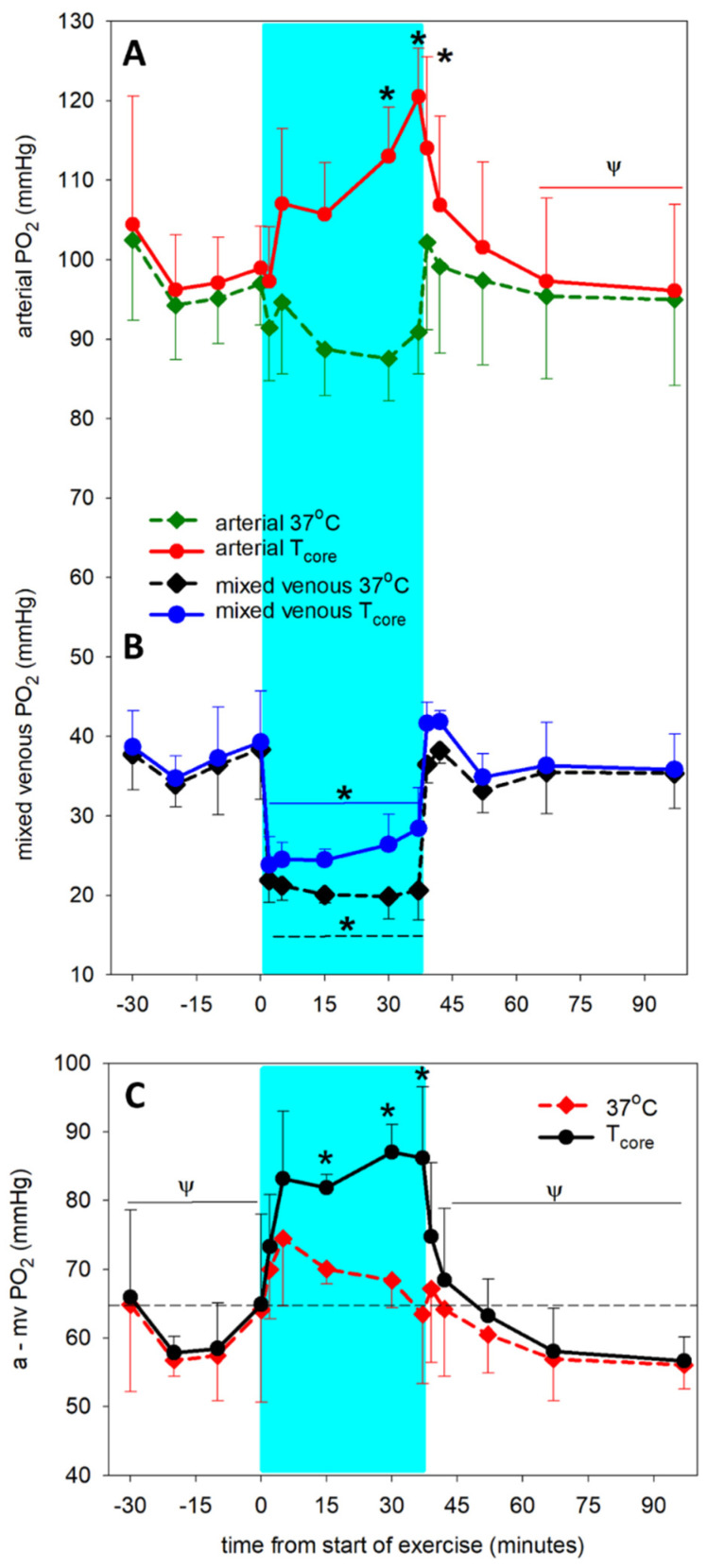
Time course of arterial (**A**) and mixed venous (mv, (**B**)) PO_2_ and a-mv PO_2_ difference (**C**) at rest prior to exercise, during exercise (shaded) and recovery from exercise. Dashed lines: measured at 37 °C. Solid lines: adjusted for core (pulmonary artery) temperature. * significantly different (*p* < 0.001) from time 0. ψ significantly different (*p* < 0.001) from end of exercise (37 min). Data from 6 horses, mean ± SD.

Plasma PO_2_ is shown for the purposes of further addressing gas exchange and demonstrating the effect of raised PA blood temperature during the exercise period. There was no change (*p* < 0.05) in arterial PO_2_ measured at 37 °C. Temperature adjustment of arterial PO_2_ significantly increased (*p* = 0.005) PO_2_ during exercise and the first 2 min of recovery (Figure 3A). In mv plasma, PO_2_ at both T_37_ and adjusted to T_core_ decreased (*p* < 0.001) by ~20 mmHg by 2 min of exercise and remained decreased until end of exercise (3B). By 2 min of recovery venous PO_2_ at both T_37_ and T_core_ had increased (*p* < 0.001) to values not different from pre-exercise. The effect of the temperature adjustment on the a-mv PO_2_ difference is significant (*p* < 0.001) during the exercise period, with the effect of increased temperature maintaining a ~10 mmHg greater a-mv PO_2_ gradient (Figure 3C).

**Figure 4:**
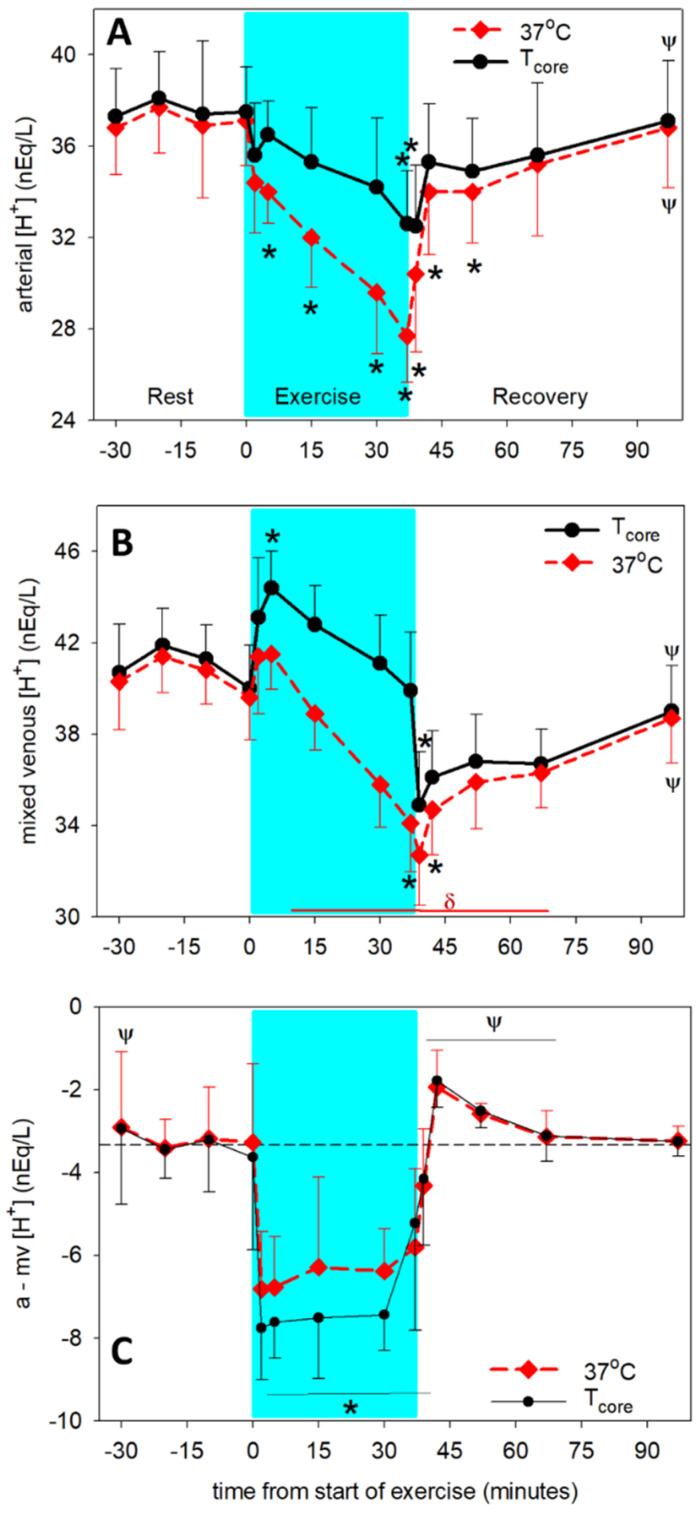
Time course of arterial (**A**) and mixed venous (mv, (**B**)) [H^+^] and a-mv H^+^] difference (**C**) at rest prior to exercise, during exercise (shaded) and recovery from exercise. Dashed lines: measured at 37 °C. Solid lines: adjusted for core (pulmonary artery) temperature. * significantly different (*p* < 0.05) from time 0. ψ significantly different (*p* < 0.05) from end of exercise (37 min). δ significantly different (*p* < 0.01) from 5 min of exercise. [H^+^] measured at 37 °C is significantly less (*p* < 0.001) than [H^+^] adjusted to core temperature during the exercise period. Data from 6 horses, mean ± SD.

The effect of temperature in the range from 37 °C to 41.5 °C on [H^+^] (Figure 4) and pH (Table 1) significantly (*p* < 0.001) increased the [H^+^]. In arterial plasma (Figure 4A), measured and temperature-adjusted [H^+^] decreased (*p* < 0.001) during the exercise period and returned to values greater (*p* < 0.001) than end-exercise by 30 min of recovery. Measured [H^+^] in mv plasma did not change during the first 5 min of exercise and then decreased (*p* < 0.001) to a nadir at 2 min of recovery, remaining below pre-exercise values until after 30 min of recovery (Figure 4B). In contrast, temperature-adjusted mv [H^+^] increased (*p* < 0.01) by 5 min of exercise then decreased significantly by the end of exercise. The a-mv [H^+^] difference became significantly more negative during exercise, and increased rapidly on cessation of exercise, reaching values not different from pre-exercise by 5 min of recovery (Figure 4C).

**Figure 5:**
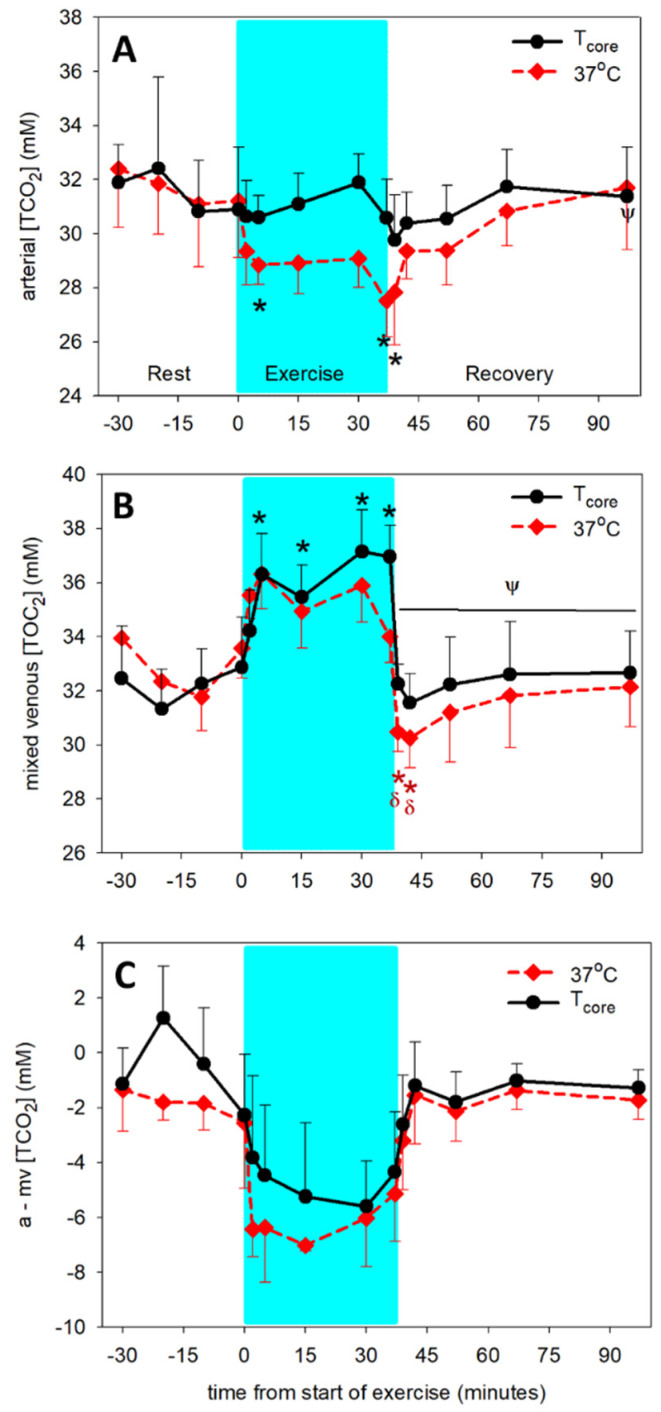
Time course of arterial (**A**) and mixed venous (mv, (**B**)) [TCO_2_] and a-mv [TCO_2_] difference (**C**) at rest prior to exercise, during exercise (shaded) and recovery from exercise. Dashed lines: measured at 37 °C. Solid lines: adjusted for core (pulmonary artery) temperature. * significantly different (*p* < 0.05) from time 0. ψ significantly different (*p* < 0.05) from end of exercise (37 min). δ significantly different (*p* < 0.01) from 5 min of exercise. Arterial [TCO_2_] measured at 37 °C is significantly less (*p* < 0.001) than [TCO_2_] adjusted to core temperature during the exercise period. Data from 6 horses, mean ± SD.

The effects of temperature on the solubility of CO_2_ in plasma, the pK′ for the dissociation of HCO_3_^−^ and the PCO_2_ resulted in increased (*p* < 0.001) arterial but not mv [TCO_2_] during exercise compared to [TCO_2_] measured at T_37_ (Figure 5). In contrast, arterial plasma [TCO_2_] measured at T_37_ decreased (*p* < 0.001) by 5 min of exercise and end of exercise, and was not different from pre-exercise during the recovery period (Figure 5A). The temperature adjusted arterial [TCO_2_] did not change throughout the exercise period and recovery. In mv plasma, [TCO_2_] at T_37_ did not change during exercise, but decreased (*p* < 0.001) rapidly below pre-exercise and end-exercise values during the first 5 min of recovery, and remained less (*p* < 0.001) than the end-exercise values through the 30 min recovery period (Figure 5B). The temperature-adjusted mv [TCO_2_] increased significantly (*p* < 0.001) during exercise and normalized within the first 2 min of recovery. Temperature adjustment of the [TCO_2_] had no effect on the a-mv [TCO_2_] difference, which remained unchanged (Figure 5C).

### 3.2. Temperature-Independent Acid-Base Variables

**Figure 6:**
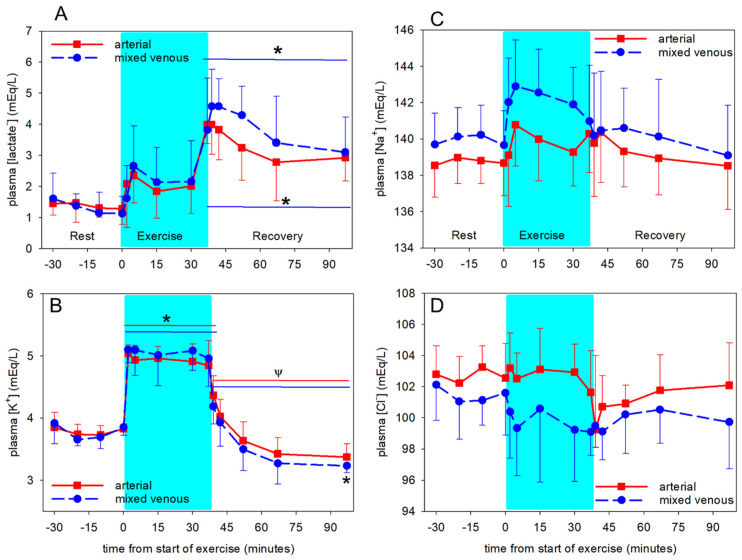
Time course of arterial (solid lines) and mixed venous (mv, dashed lines) plasma [lactate^−^] (**A**), [K^+^] (**B**), [Na^+^] (**C**) and [Cl^−^] (**D**) at rest prior to exercise, during exercise (shaded) and recovery from exercise. * significantly different (*p* < 0.05) from time 0. ψ significantly different (*p* < 0.05) from end of exercise (37 min). Data from 6 horses, mean ± SD.

[Lactate^−^] in both arterial and mv plasma was not increased until the end of exercise and then remained greater than pre-exercise throughout 60 min of recovery (Figure 6A). Arterial [lactate^−^] was less (*p* < 0.001) than in mv plasma during exercise and recovery (Figure 6A). There was no change in a-mv plasma [lactate^−^] during the entire experiment (not shown). Arterial and mixed venous plasma [K^+^] increased (*p* < 0.001) within 2 min of exercise and remained constant and elevated throughout exercise (Figure 6B). Plasma [K^+^] rapidly decreased (*p* < 0.001) within 2 min of recovery and was lower (*p* < 0.001) than pre-exercise by 15 min of recovery. Venous [K^+^] was greater than arterial [K^+^] during exercise (*p* = 0.044) and lower than arterial during the last 15 min of recovery (*p* = 0.038), but there was no significant change in a-mv [K^+^] during the entire experiment (data not shown). Repeated measures ANOVA indicated significant changes over time for arterial plasma [Na^+^] (*p* = 0.015; Figure 6C) and [Cl^−^] (*p* = 0.014; Figure 6D), but the post hoc test did not identify any differences between means. However, mv [Na^+^] was greater (*p* < 0.001) than arterial, and was increased (*p* < 0.001) at 2, 5 and 15 min of exercise compared to pre-exercise. Additionally, mv [Cl^−^] was less (*p* < 0.001) than arterial (Figure 6C,D). There were no significant changes in a-mv differences for [Na^+^] and [Cl^−^] (data not shown).

**Figure 7:**
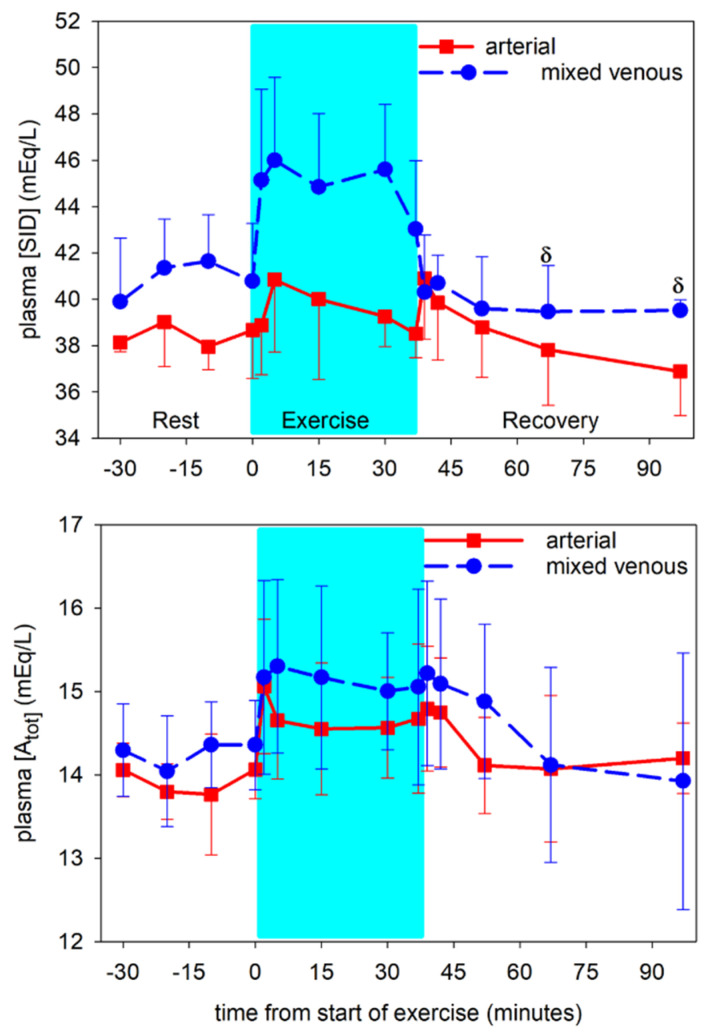
Time course of plasma [SID] (**top**) and [A_tot_] (**bottom**) in arterial (solid lines) and mixed venous (dashed lines) blood at rest prior to exercise, during exercise (shaded) and recovery from exercise. δ significantly different (*p* < 0.01) from 5 min of exercise. Data from 6 horses, mean ± SD.

Arterial plasma [SID] was variable and unchanged (*p* = 0.288) throughout the experiments. In contrast, mv [SID] was increased (*p* < 0.01) at 5 min of exercise compared to pre-exercise and the entire exercise period, then returned to pre-exercise values within 2 min of recovery (Figure 7). The increase in mv [SID] was due to both increased mv [Na^+^] and decreased mv [Cl^−^] (Figure 6).

Arterial [A_tot_] was less than (*p* < 0.001) mv [A_tot_] at all except the final time point (Figure 7). Arterial [A_tot_] increased (*p* = 0.011) at 2 min of exercise compared to pre-exercise, and was variable thereafter and not significantly different from pre-exercise during the rest of exercise and recovery. Venous [A_tot_] was increased at 5 min of exercise compared to pre-exercise (*p* = 0.029). Arterial and mv [TP] mirrored the [A_tot_] data (Table 1).

There was no difference between arterial and venous Hct throughout the time course (Table 1). Both arterial and venous Hct increased (*p* < 0.001) by 2 min of exercise and remained constant and elevated until the end of exercise. Hct decreased after cessation of exercise and was lower (*p* < 0.001) than the end-exercise values by 10 min of recovery.

### 3.3. Determining the Origins of the Changes in Dependent Acid-Base Variables

**Figure 8:**
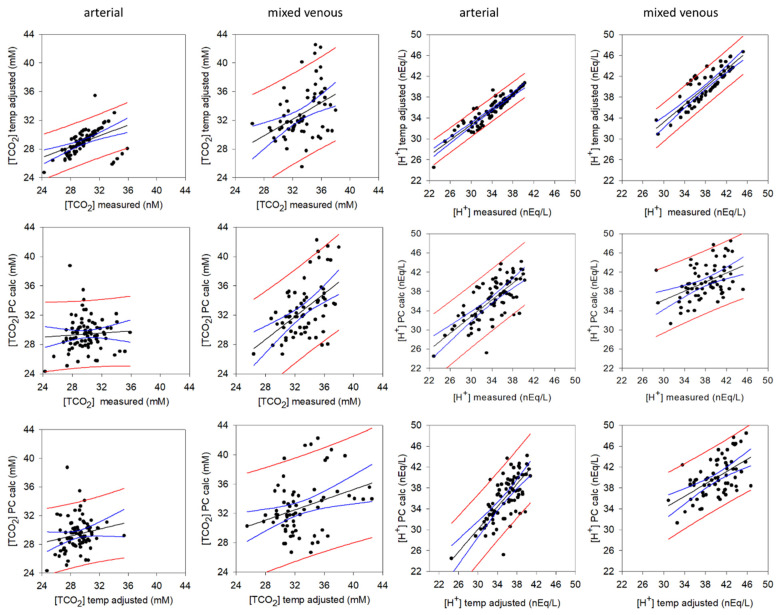
Results of the linear regression analyses relating measured, calculated and temperature-adjusted [TCO_2_] and [H^+^] in arterial plasma and mixed venous plasma. The equations and coefficients are presented in Table 2. Straight black line: predicted values; curved blue lines: 95% confidence interval; red lines: standard error.

When determining the origins of the changes in the dependent acid-base variable, it is useful to demonstrate agreement between “measured” variables and the “calculated” variables. The “measured” variables are those reported by the instrument at 37 °C, the temperature-adjusted values for [H^+^] and [TCO_2_] are with respect to measured pulmonary artery temperature at each time point, and the “calculated” (PC calc) values are those calculated using the ACID-BASICS software from the [SID]. The relationships are shown in Figure 8 and the regression details provided in Table 2. Measured versus calculated [H^+^] were highly correlated (*p* < 0.001) for both arterial and mv samples. Measured vs. calculated arterial [TCO_2_] were not strongly correlated (*p* > 0.001), whereas measured and calculated mv [TCO_2_] were highly correlated (*p* < 0.001). The inherent variability in these relationships arises from effects of temperature as well as the variability in each of the 6 independent measures used in their calculation: PCO_2_, [A_tot_], [Na^+^], [K^+^], [Cl^−^] and [lactate^−^].

The contributions of changes in each of the independent variables to the [H^+^] and [TCO_2_] were calculated based on using the Stewart program calculated values, but with temperature-adjusted PCO_2_. These values therefore differ somewhat from measured values, but this is necessary when calculating the contributions of each independent variable.

**Figure 9:**
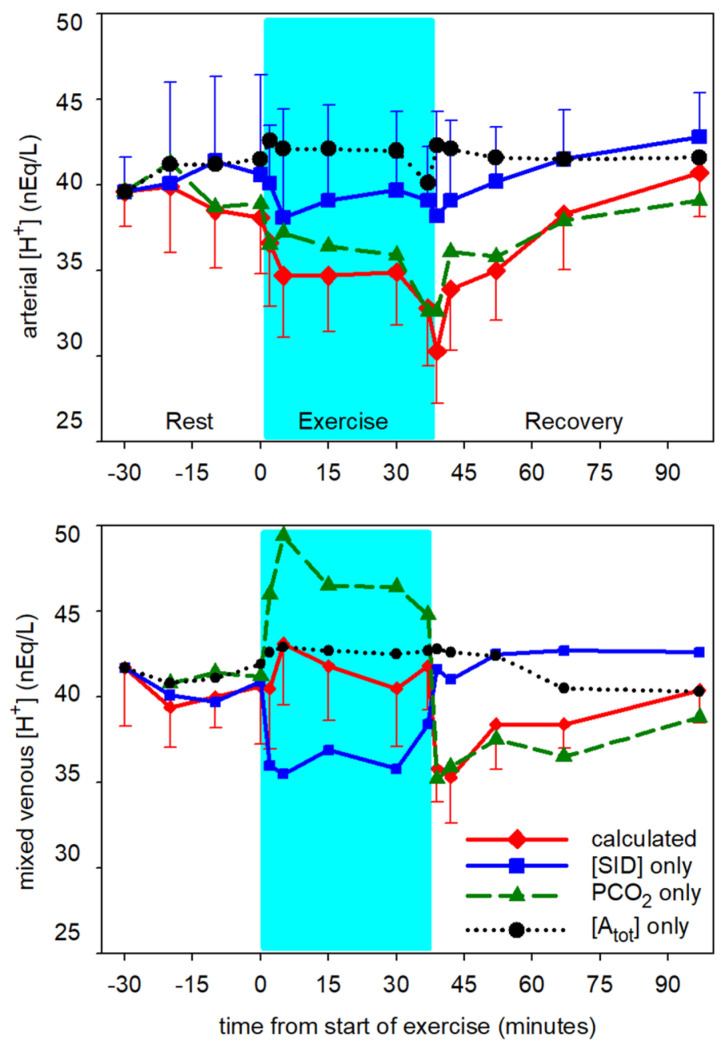
Time course of contributions to change in calculated [H^+^] from the independent acid-base variables in arterial (**top**) and mixed venous (mv, **bottom**) plasma at rest prior to exercise, during exercise (shaded) and recovery from exercise. Data from 6 horses, mean ± SD. Error bars omitted are similar in magnitude to those presented for another variable. Arterial: [A_tot_] and [SID] contributions significantly different (*p* < 0.001) from calculated [H^+^] during exercise and recovery. mv: PCO_2_ and [SID] contributions significantly different (*p* < 0.001) from calculated [H^+^] during exercise and recovery.

With respect to [H^+^], this level of moderate intensity exercise resulted in a mild arterial alkalosis (Figure 9 top). The primary contributor to the decrease in arterial [H^+^] was the decrease in PCO_2_, and secondarily the increase in [SID]. The alkalizing effects of these two changes were slightly offset by the increase in [A_tot_], which contributes to raising [H^+^]. There was no change in calculated mv [H^+^] during the exercise period (Figure 9 bottom). The rapid increase in mv PCO_2_ had a strong acidifying effect, while the increase in [A_tot_] had a secondary contribution to acidosis with the onset of exercise; these acidifying effects were completely offset by the increase in mv plasma [SID]. In the first 15 min of recovery, the pronounced decrease in venous PCO_2_ contributed nearly 100% to the post-exercise decrease in [H^+^], as [SID] and [A_tot_] returned to pre-exercise levels.

**Figure 10:**
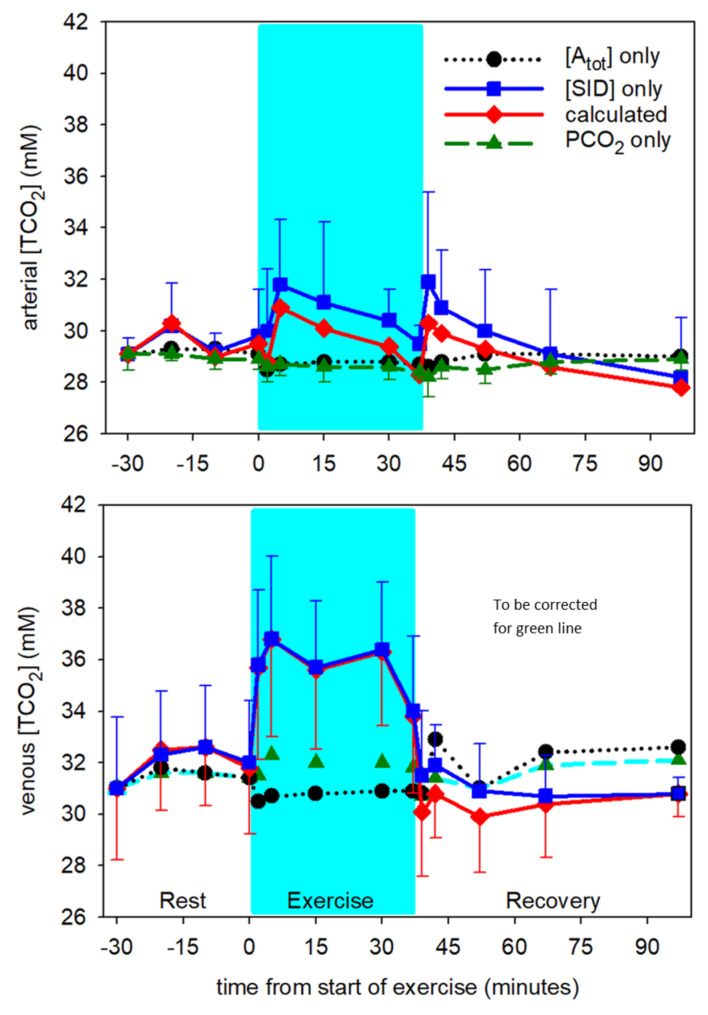
Time course of contributions to change in calculated [TCO_2_] from the independent acid-base variables in arterial (**top**) and mixed venous (mv, **bottom**) plasma at rest prior to exercise, during exercise (shaded) and recovery from exercise. Data from 6 horses, mean ± SD. Error bars omitted are similar in magnitude to those presented for another variable. Arterial: [A_tot_] and PCO_2_ contributions significantly different (*p* < 0.001) from calculated [H^+^] during exercise. mv: [A_tot_] and PCO_2_ contributions significantly different (*p* < 0.001) from calculated [H^+^] during exercise.

While the changes in arterial [TCO_2_] were not statistically significant, the contributions of independent variables were still apparent for both arterial and venous [TCO_2_] (Figure 10). A rapid increase in arterial and venous [TCO_2_] with onset of exercise was completely attributed to the increase in [SID] at the onset of exercise and through the exercise period. The contributions of PCO_2_ and [A_tot_] to the [TCO_2_] were negligible through exercise and recovery.

## 4. Discussion

**Figure 11:**
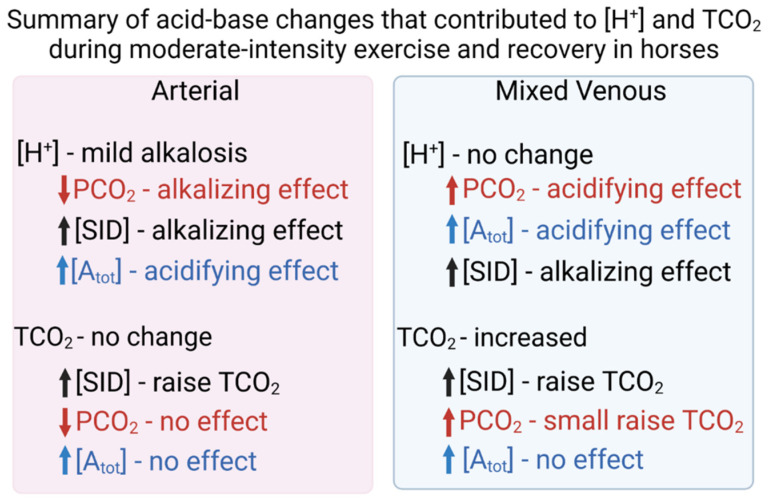
Summary of the main independent variable origins (contributions) to arterial and mixed venous plasma [H^+^] and [TCO_2_] during moderate intensity exercise in horses.

The present paper presents the first detailed physicochemical analysis of both arterial and mv blood plasma in horses performing moderate intensity exercise to the point of clinical hyperthermia (T_core_ 41.5 °C). By integrating the physicochemical approach with adjustment of temperature-dependent plasma gas and acid-base variables to core body temperature, the present study demonstrated a mild exercise-induced alkalosis in both arterial and mv plasma, albeit of lesser magnitude in mv than arterial plasma. The alkalizing effect of increased [SID] in mv plasma offset the acidifying effects of increased PCO_2_ and [A_tot_] (Figure 11). In arterial plasma, it was the alkalizing effect of reduced PCO_2_ that was the primary contributor to the alkalosis. The effect of temperature during exercise was insignificant for pH; however, increased T_core_ resulted in increased PCO_2_, [TCO_2_], [HCO_3_^−^] and PO_2_ during the exercise period only. Temperature-induced increases in PCO_2_, [TCO_2_] and [HCO_3_^−^] had no effect on their a-mv concentration differences. However, the temperature induced increase in PO_2_ increased the a-mv PO_2_ difference during exercise, thus increasing the gradient for O_2_ flux from the arterial circulation to the contracting muscles.

### 4.1. Temperature Adjustment

Previous studies that have applied Stewart’s physicochemical approach to acid-base analysis in exercising horses [10,11,12,13,39,47], as well as descriptive acid-base studies [4,5,48,49] have not applied temperature adjustments. High intensity exercise in horses results in large increases in core and peripheral temperatures [33,49,50,51], with core temperatures in excess of 43 °C reported at the end of high intensity exercise conducted in hot, humid conditions [49]. While effects of temperature over this temperature range on plasma pH are relatively small, the effect of the increase in temperature on the partial pressures of gases [32] and hence on the [HCO_3_^−^] and [TCO_2_] [52,53] are large (Figure 5 and Figure 6). The effects of increasing temperature during exercise contributed to a rapid increase in venous PCO_2_ to values two-fold greater than measured at T_37_, and venous PCO_2_ was maintained above pre-exercise values until cessation of exercise (Figure 3B). Thus, in vivo, there was no venous hypocapnia as was suggested by PCO_2_ at T_37_. In addition, the effect of temperature also largely abolished the arterial hypocapnia seen at T_37_, with significant hypocapnia seen only at the very end of exercise (Figure 3A). In contrast to the arterial PCO_2_, the arterial PO_2_ at TPA increased substantially during exercise (Figure 4A) driven by both temperature effects and the hyperpnea of exercise. Both the temperature effect and the hyperpnea [54] would augment oxygen delivery to contracting muscles.

### 4.2. Gas Exchange

When the arterial and mixed venous PCO_2_ and PO_2_ are considered in light of Forster’s work on control of breathing in ponies performing submaximal exercise [34,47,54,55,56] it becomes evident that the effects of temperature on control of breathing should be appreciable. The time course of changes in PCO_2_ and PO_2_ (at T_37_) in the present study agree with that of Forster. In Forster’s studies, because arterial and mixed venous PCO_2_, as well as [H^+^], decreased in the first minutes of exercise it was concluded that neither delivery of CO_2_ to the lungs nor the arterial PCO_2_ provided primary stimuli for the hyperpnea of exercise [56,57]. In contrast, the decrease in mixed venous PO_2_ with exercise was immediate (Figure 4B) and neural feed-forward (central command) preceded onset of exercise, supporting the concept that stimuli originating in exercising limbs and conveyed to the brain by spinal afferents contribute to the exercise hyperpnea [58] and thus account for the time course of changes in arterial PCO_2_ and PO_2_.

The onset of exercise results in an increase in oxygen extraction (Figure 4B) and CO_2_ release (Figure 3B) by contracting muscles. The resultant increase in venous PCO_2_ was due to both increased CO_2_ production (seen by the increase in venous PCO_2_ at T_37_) as well as the increase in temperature, which has a profound effect on increasing the solubility and partial pressure of blood gases [29,32]. Temperature effects on the muscle are greater than at the core [37,51], which is beneficial for both oxygen extraction (Figure 4B) and CO_2_ removal because higher partial pressure gradients are present. Temperature adjusted mixed venous PCO_2_ was ~60 mmHg, which means that the venous PCO_2_ leaving contracting muscles was likely 10 to 20 mmHg greater given the tissue mass and blood flow involved.

Perfusion of the lungs with mixed venous blood results in a decrease in blood temperature (unless ambient temperatures are hot), release of CO_2_ and uptake of O_2_. In the present study, it is clear that arterial PCO_2_ is involved in the control of breathing because the hyperventilation of exercise significantly reduced the PCO_2_ (Figure 3A). In contrast, the arterial PO_2_ was essentially maintained (Figure 4A). With exercise in equids, the drive to ventilate is not mediated by central chemoreceptors, as ventilation is elevated within seconds of initiation of exercise [37,59]. Increased arterial [H^+^] and PCO_2_ within the first 5 min of exercise were small and short-lasting, and not likely of physiological significance. The early drive to ventilate has been shown to be mediated by peripheral receptors associated with increased muscle PCO_2_ and central neural drive [54,58].

### 4.3. Factors Contributing to the Acid-Base Changes of Moderate-Intensity Exercise

The present study used the physicochemical acid-base approach to determine or partition the contributions of each of the independent acid-base variables ([SID], pCO_2_, and the [A_tot_]) in plasma to the changes in dependent acid-base variables ([H^+^], [HCO_3_^−^], [TCO_2_]) as detailed in only a few previous studies [11,25,38,39]. These types of interpretations furthered those presented by Forster and colleagues more than 30 years ago [34,47].

The physicochemical approach detailed by Stewart is based on the early research [22,60] that show that increases in PCO_2_ have an acidifying effect, increases in the concentrations of strong base cations (Na^+^ and K^+^, mainly) have an alkalizing effect, increases in the concentrations of strong acid anions (Cl^−^ and lactate, mainly), and increases in the total concentrations of weak acids, i.e., albumin and phosphate, will have an acidifying effect [23]. The intensity of exercise in Forster et al.’s acid-base study at ~50% of maximal for ponies [34] appeared to be similar to that of the present study (~50% of maximal heart rate) based on the magnitude of changes in measured variables during the first 5 min of exercise. In both studies, both arterial and mixed venous [H^+^] decreased during exercise, and arterial [HCO_3_^−^] decreased while venous [HCO_3_^−^] tended to increase. When interpreting the causes of the changes in [H^+^] and [HCO_3_^−^], Forster et al. did not calculate what the dependent acid-base variables would have been with a change in only one of the three independent variables. Nonetheless, their interpretation of the decrease in arterial plasma [H^+^] to be primarily due to a decreased PCO_2_ and secondarily to increased [SID] is completely consistent with detailed analysis performed in the present study (Figure 7). A difference between the two studies is in the analysis of mixed venous plasma, which Forster and coworkers did not consider. In the present study, the increased mixed venous PCO_2_ had a pronounced acidifying effect, but this was completely negated by the simultaneous increase in [SID] (Figure 7). In both arterial and venous plasma, the increase in [A_tot_] is mainly due to the increase in plasma protein concentration that results from a net movement of protein-poor fluid into contracting muscles [61,62]. This weak acid contribution to plasma acidification was small and did not contribute significantly to the acid-base changes.

With high intensity exercise, the contributions of the independent acid-base variable to changes in [H^+^] and [HCO_3_^−^] or [TCO_2_] are very different from what is seen at moderate intensity exercise. With high intensity exercise, there are marked increases in [H^+^] and decreases in [HCO_3_^−^] and [TCO_2_] [10,33,39,49]. Two minutes of high intensity trotting by standardbred racehorses showed, using jugular venous blood, that a decreased [SID] was primarily responsible and increased [A_tot_] secondarily responsible for the plasma acidosis, while the decreased PCO_2_ contributed a mild alkalizing effect [39]. While Vengust et al. [10] determined the physicochemical variables with high intensity (~80% of peak VO_2_), they did not report their concentrations in plasma and also did not determine the contributions of independent variables to the dependent acid-base variables. Examination of their data also demonstrates that there was no decrease in arterial PCO_2_ until cessation of exercise, i.e., at a time point commensurate with the first post-exercise measurement of Waller and Lindinger [39].

Low to moderate intensity exercise results in a reduction in [H^+^]_a_ and a decrease in P_a_CO_2_ [33,55,63,64]. With higher exercise intensity, there is an increase in [H^+^]_a_, with the increase proportional to the intensity of the exercise [10,39,56,64,65]. In Pan et al.’s study [56], the exercise duration was limited to 6 min, followed by 6 min of recovery.

Exercise duration in horses is limited compared to humans because of the high rate of body heat storage and rapid achievement of pathologically high tissue temperatures [51]. Taylor et al. [33] studied acid-base responses in Arabian breed horses, a breed suited to longer durations of exercise and with a body mass to surface area ratio that better facilitates heat dissipation compared to mature standardbred and thoroughbred horses. The horses in Taylor’s study exercised for a total duration of 52 min, with 13 small increments (speed increase of 0.5 m/s at constant slope of 6% [3.6°]) in intensity at 4 min intervals, thus achieving (near) steady-state at the end of each increment. Thus, the intensity at the end of exercise was still “moderate”. In this study, [H^+^]_a_ fell during the first 10 min of exercise, and was stable for the next three increments, then decreased sharply with the next two increments, followed by a gradual increase for the remainder of the exercise period despite increasing intensity. [H^+^]mv remained unchanged for the first 26 min, followed a small decrease, then a continuing increase to above resting at the end of exercise.

## 5. Limitations

While the present study is descriptive, it tries to provide a mechanistic basis for the changes in arterial and mixed venous blood acid-base state. As with previous studies [10,11,12,39,47], the present study is limited primarily by the variability surrounding the measurement of each individual variable that contributes to the acid-base state, and secondarily by the small number of animals studied. Using larger numbers of animals would not affect the variability, as acid-base balance in horses is inherently variable [66]. A third limitation is that temperature adjustments were applied using core body (PA) temperature, while temperatures in contracting muscles at the end of exercise approached 43 °C while peripheral temperatures only approached 39 °C [39]. Measurement variability can make it difficult to identify clear responses when the physicochemical approach is applied to small groups of animals because of the animal variability, in contrast to when the approach is used with individuals [13,28]. The animal variability reflects both the number of individual plasma variables that change as well as differences in strategies used to control each of the plasma variables over time [28]. While this is unavoidable, it is necessary to take great care to obtain the most accurate measures of blood variables possible.

## 6. Conclusions

The significance of the present study is that correction of acid-base variables to, in this case, core body temperature presents a markedly different physiological response to exercise than that provided by variables measured and presented at an instrument temperature of 37 °C. Even with moderate intensity exercise in thermoneutral conditions, the resultant increase in body temperature affected acid-base status and gas partial pressures. The site of blood sampling is also important: a mild alkalosis had markedly different origins in arterial blood than in mixed venous blood. In mixed venous blood, increased CO_2_ had a major acidifying effect that was completely offset by increased [SID]. In arterial blood, decreased CO_2_ had an alkalizing effect that was offset by increased [A_tot_]. In order to fully understand how acid-base status changes during exercise and recovery, it is importance to quantify the changes in both arterial and mixed venous blood, with adjustment to core temperature.

## Figures and Tables

**Table 1:** Measured independent acid-base variables, and measured and calculated dependent acid-base variables (pH and [HCO_3_^−^]) and hematocrit.

Arterial	pH_meas_ Mean	SD	pH_adj_ Mean	SD	pH_calc_ Mean	SD	[HCO_3_^−^]_m_ Mean	SD	[HCO_3_^-^]_adj_ Mean	SD	[HCO_3_^−^]_calc_ Mean	SD	[TP] Mean	SD	HCT (%) Mean	SD
−30	7.435	0.0364	7.430 **	0.0364	7.403	0.0225	30.96 **	2.03	28.68	1.34	27.71	0.53	6.28	0.14	37.40 **	3.91
−20	7.425	0.0239	7.420 **	0.0239	7.403	0.0609	30.47 **	1.81	29.17	2.19	28.78	1.40	6.16	0.15	34.50 **	5.28
−10	7.434	0.0388	7.429 **	0.0388	7.417	0.0500	29.73 **	2.14	27.67	1.77	27.66	1.19	6.15	0.22	34.67 **	5.89
0	7.431	0.0233	7.426 **	0.0233	7.42	0.0379	29.83	2.02	27.74	2.17	28.12	1.90	6.28	0.16	38.67	5.09
2	7.464	0.0283	7.449	0.0283	7.439	0.0428	28.13	1.22	27.49	1.25	27.47	1.91	6.73 *	0.36	49.5 *	5.47
5	7.468	0.0182	7.438	0.0178	7.462	0.0462	27.67	0.74	27.47	0.76	29.60	3.06	6.54	0.31	51.3 *	5.57
15	7.496 *	0.0294	7.453	0.0289	7.462	0.0538	27.80	1.20	27.93	1.07	28.83	2.90	6.50	0.35	48.7 *	3.78
30	7.53 *	0.0387	7.468 *	0.0377	7.46	0.0501	28.05	1.01	28.68	1.01	28.09	0.88	6.50	0.27	48.5 *	3.94
37	7.559 *	0.0317	7.488 *	0.0309	7.486	0.0441	26.62 *	1.34	27.44	1.35	27.11	1.21	6.55	0.40	47.8 *	2.59
39	7.521 *	0.0369	7.492 *	0.0359	7.523 *	0.0729	26.817 *	1.77	26.66	1.58	29.17	4.67	6.61	0.33	48.5 *	2.26
42	7.47	0.0318	7.453	0.0315	7.464	0.0429	27.83	1.17	27.26	1.08	28.59	2.10	6.52	0.31	46.0 *	2.37
52	7.469	0.0296	7.458	0.0293	7.457	0.0359	28.17	1.20	27.41	1.16	27.98	2.12	6.30	0.26	41.50 **	2.88
67	7.455	0.0388	7.45	0.0288	7.419	0.0494	29.53 **	1.25	28.54	1.30	27.28	2.14	6.28	0.39	39.67 **	3.14
97	7.435	0.0319	7.432 **	0.0319	7.391 **	0.0278	30.32 **	2.14	28.19	1.72	26.43	1.85	6.34	0.19	37.83 **	3.92
Venous																
−30	7.396	0.0348	7.391	0.0348	7.381	0.0352	32.63	1.46	29.22 **	2.77	29.44	2.73	6.38	0.25	36.50 **	4.04
−20	7.383	0.0168	7.378	0.0168	7.405	0.0262	31.17	1.15	28.15 **	2.33	30.918	2.287	6.27	0.297	34.75 **	4.11
−10	7.39	0.0157	7.385	0.0157	7.398	0.0194	30.6	1.06	29.03	1.21	31.017	2.234	6.41	0.23	38.25 **	5.19
0	7.403	0.0205	7.398	0.0205	7.393	0.0368	32.32	2.06	29.60 **	1.756	30.203	2.559	6.41	0.24	38.40 *	5.08
2	7.383	0.0265	7.366 **	0.0265	7.394	0.0366	34.08	1.26	30.88	3.318	33.94	4.40	6.77	0.53	50.7 *	5.79
5	7.382	0.0164	7.353 *	0.0161	7.367	0.0464	32.9	2.41	32.86	2.359	34.91	3.77	6.83	0.46	50.6 *	4.83
15	7.41 **	0.0178	7.369 **	0.0178	7.381	0.0533	33.7	2.52	32.06	3.015	33.76	5.01	6.77	0.49	47.8 *	3.27
30	7.447 *	0.023	7.386	0.0226	7.394	0.0366	34.68	1.45	33.65 *	2.391	34.53	2.85	6.70	0.31	48.4 *	2.70
37	7.469 *	0.0424	7.401	0.0414	7.379	0.0272	32.64	0.97	33.47 *	1.091	32.03	2.89	6.72	0.52	49.4 *	2.51
39	7.487 *	0.0304	7.458 *	0.0301	7.447	0.0232	29.28	0.69	29.01 **	0.692	28.73	2.43	6.79	0.49	47.6 *	2.61
42	7.46 *	0.0256	7.443 *	0.0256	7.447	0.0415	29.04	1.05	28.35 **	1.025	29.22	1.56	6.74	0.45	44.8	2.59
52	7.445 *	0.025	7.435 *	0.0245	7.417	0.0414	29.88	2.69	28.99 **	2.60	28.47	2.06	6.64	0.41	40.8 **	3.49
67	7.44 **	0.0184	7.435	0.0184	7.417	0.0158	30.43	1.87	29.35 **	1.854	28.91	2.03	6.31	0.52	38.8 *	1.89
97	7.412 **	0.0225	7.41	0.0225	7.394	0.0204	30.63	1.48	29.41 **	1.456	29.25	0.93	6.212	0.69	38.0 *	3.92

* significantly different (*p* < 0.05) from time 0; ** significantly different (*p* < 0.05) from end exercise (t = 37 minutes of exercise).

**Table 2:** Results of the linear regression analyses between measured and calculated [H^+^] and [TCO_2_] for the graphs presented in Figure 8.

[H^+^]	*n*	R^2^	SEE	*p*
Arterial				
[H^+^] temp adj = 0.697 + (0.982 × [H^+^] meas)	82	1.000	0.053	<0.001
[H^+^] ion stewart = 6.759 + (0.867 × [H^+^] meas)	82	0.517	3.217	<0.001
[H^+^] ion stewart = 6.068 + (0.886 × [H^+^] temp adj)	82	0.520	3.208	<0.001
Mixed venous				
[H^+^] temp adj = 0.522 + (0.987 × [H^+^] meas)	68	1.000	0.048	<0.001
[H^+^] stewart = 22.108 + (0.469 × [H^+^] meas)	68	0.208	3.269	<0.001
[H^+^] stewart = 21.734 + (0.479 × [H^+^] temp adj)	68	0.211	3.263	<0.001
TCO_2_				
Arterial				
TCO_2_ temp adj = 17.746 + (0.377 × TCO_2_ meas)	82	0.219	1.516	<0.001
TCO_2_ stewart = 27.334 + (0.0693 × TCO_2_ meas)	82	0.00418	2.272	0.564
TCO_2_ stewart = 22.390 + (0.242 × TCO_2_ temp adj)	82	0.0331	2.239	0.102
Mixed venous				
TCO_2_ temp adj = 13.549 + (0.582 × TCO_2_ meas)	68	0.172	3.148	<0.001
TCO_2_ = 0.00678 + (0.000782 × TCO_2_ meas)	68	0.271	0.003	<0.001
TCO_2_ stewart = 0.0153 + (0.000522 × TCO_2_ temp adj)	68	0.381	0.003	<0.001

meas = measured at instrument temperature of 37 °C; temp adj = pco_2_ adjusted to measured blood temperature; stewart = calculated using the ACID-BASICS software.

## Data Availability

The data that support the findings of this study are available from the corresponding author upon reasonable request.
